# Distribution of Bacterial Blight Resistance Genes in the Main Cultivars and Application of *Xa23* in Rice Breeding

**DOI:** 10.3389/fpls.2020.555228

**Published:** 2020-08-31

**Authors:** Shiguang Wang, Wei Liu, Dongbai Lu, Zhanhua Lu, Xiaofei Wang, Jiao Xue, Xiuying He

**Affiliations:** ^1^Rice Research Institute, Guangdong Academy of Agricultural Sciences, Guangzhou, China; ^2^Guangdong Key Laboratory of New Technology in Rice Breeding, Guangzhou, China

**Keywords:** rice, bacterial blight, distribution, *Xa23*, marker-assisted selection

## Abstract

Bacterial blight (BB) is an important constraint on achieving a high and stable rice grain yield. An increasing number of BB resistance (*R*) genes have been identified and cloned to increase the available options for rice disease resistance breeding. However, it is necessary to understand the distribution of *R* genes in rice varieties for rational distribution and breeding. Here, we genotyped five *R* genes, *i.e. Xa4*, *Xa7*, *Xa21*, *Xa23*, and *Xa27*, in seventy main cultivars from Guangdong Province, South China using the corresponding specific markers. Our results showed that 61 varieties carried *Xa4*, only three varieties carried *Xa27*, and *Xa7*, *Xa21*, or *Xa23* was not detected in all tested varieties. Notably, only 33 varieties exhibited resistance to pathotype IV *Xoo* strains. These results indicate that *Xa4* is no longer suitable for widespread use in rice breeding, although *Xa4* is widely present in tested varieties. Remarkably, the strongly virulent BB strains of pathotype IX evolved quickly in southern China, and *Xa23* was found to effectively confer resistance against the pathotype IX strains. Subsequently, we successfully bred two novel inbred rice varieties as also being restorer lines and two photoperiod- and thermo-sensitive genic male sterility (P/TGMS) lines using the broad-spectrum resistance gene *Xa23* through marker-assisted selection (MAS) combined with phenotypic selection. All of the developed lines and derived hybrids exhibited enhanced resistance to BB with excellent yield performance. Our research may potentially facilitate both of the inbred and hybrid rice disease resistance breeding.

## Introduction

Rice is one of the most important staple crops because it feeds nearly half of the global population. The grain production and quality of rice have to be increased and improved to guarantee global food security due to the rapid growth of the global population, global climate change, and other reasons (Tilman et al., 2001; Qian et al., 2016). However, rice grain yield and quality are frequently and severely threatened by devastating diseases caused by multiple pathogens, such as *Xanthomonas oryzae* pv. *oryzae* (*Xoo*) and *Magnaporthe oryzae* (Ke et al., 2017; Wang J. et al., 2018). Previous studies have shown that significant grain yield loss of more than 20% was caused by BB, especially in hybrid rice, and the degree of yield loss was affected by the stage of BB infection (Chukwu et al., 2019). Therefore, the breeding of resistant rice varieties is advocated as being the most cost-effective approach to overcome the major obstacle to achieving optimal yield. Consequently, the exploration of novel resistant germplasms and the utilization of excellent gene resources are crucial for the development of rice resistance to BB.

To date, approximately 45 resistance (*R*) genes conferring resistance against BB have been identified using diverse rice sources (Neelam et al., 2020). However, only eleven of these *R* genes, exhibiting multiple mechanisms of *R*-gene-mediated *Xoo* resistance, have been cloned and functionally analyzed (Song et al., 1995; Yoshimura et al., 1998; Iyer and McCouch, 2004; Sun et al., 2004; Gu et al., 2005; Chu et al., 2006; Liu et al., 2011; Tian et al., 2014; Hutin et al., 2015; Wang et al., 2015; Hu et al., 2017). Among these genes, *Xa21*, *Xa23*, and the un-cloned *R* gene *Xa7* act in a dominant manner and confer the strongest resistance to BB with the broadest resistance spectrum (Zhang, 2009; Chukwu et al., 2019). Recently, many *R* genes for BB were successfully incorporated into both elite inbred varieties and parental lines of hybrid rice to control the disease using MAS (Chukwu et al., 2019; Jiang et al., 2020; Li W. et al., 2020). Notably, a few of those *R* genes, such as *Xa3*, *Xa4*, *Xa7*, and *Xa21*, have been widely utilized in rice resistance breeding since the 1980s (Deng Q. et al., 2006; Zhang, 2009; Chen et al., 2011; Luo et al., 2012). However, the resistance conferred by these genes has weakened and can be overcome by the new virulent BB strains due to pathogenic variation or evolution with consequent breakdown of rice resistance (Zhang, 2009; Chen et al., 2011; Wang et al., 2014). On the other hand, the *R*-gene-mediated resistance to *Xoo* in inbred rice or hybrid rice may be influenced by the rice genetic background and the incomplete dominance of *R* genes, thus hindering the application of these *R* genes for breeding of disease-resistant rice (Luo et al., 2012; Zeng L. et al., 2017). Remarkably, the resistance gene *Xa23*, a new executor *R* gene derived from wild rice (*Oryza rufipogon*), was found to effectively confer completely dominant and broad-spectrum resistance against BB of rice at all growth stages (Wang et al., 2015). Thus, CBB23, a near isogenic line (NIL) carrying the *Xa23* resistance haplotype in the JG30 background, has been widely utilized as an important germplasm for disease resistance in rice breeding (Zhang, 2009; Wang et al., 2015).

There is high selection pressure due to the high cropping index in the two-season continuous-cropping rice area and the specific ecological conditions, such as high temperature with high humidity, in southern China, which facilitates pathogenic variation and results in epidemics of BB (Chen et al., 2018). Thus, the strongly virulent BB strains of pathotype V have replaced pathotype IV strains and have developed into the preponderant pathotype, and the more virulent pathotype IX has grown quickly in southern China (Chen et al., 2018). On the other hand, there have been few studies on the rationality of the distribution of rice varieties resistant to BB, whereas varieties carrying a single *R* gene are subjected to large-scale and long-term cultivation, resulting in breakdown of resistance (Kim, 2018; Chukwu et al., 2019). In the present study, we identified the distribution of the BB resistance genes of the main cultivars from Guangdong Province, China, for varieties that are rationally distributed and bred. We found that *Xa4* was widely utilized, while other detected *R* genes were less frequently used for rice breeding in Guangdong. Based on this finding, the underlying objective of this study was to breed new rice varieties and novel P/TGMS lines using the broad-spectrum resistance gene *Xa23*, which conferred high resistance to all of the tested *Xoo* strains, including the strongly virulent BB strains of pathotype V and IX (Luo et al., 2013). Finally, two novel inbred rice varieties and two newly developed P/TGMS lines with significantly enhanced disease resistance to BB were successfully bred using MAS combined with phenotypic selection. The improved lines and the derived hybrid combinations with elite yield performance showed high resistance to the tested *Xoo* strains, and provided excellent paternal lines for production of inbred and hybrid rice with disease resistance to rice BB.

## Materials and Methods

### Plant Materials and Growth Conditions

**Figure 1:**
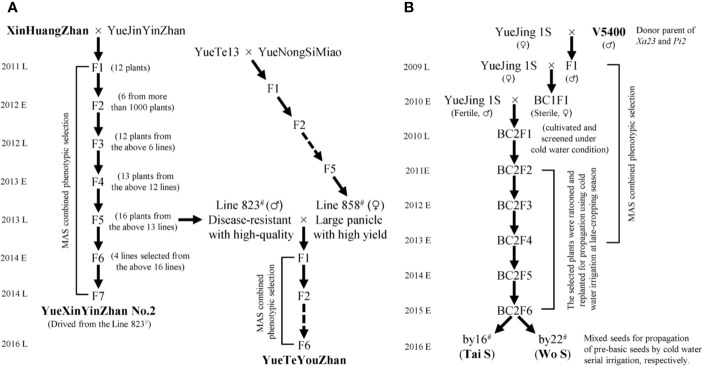
Flowchart for the development of disease-resistant varieties and P/TGMS lines. MAS, marker-assisted selection; E and L represent the early cropping season and later cropping season, respectively. **(A)** The pedigree breeding schemes for the inbred lines. **(B)** The backcross breeding schemes for the P/TGMS lines.

Seventy main cultivars with a planting area of more than 6,000 ha in Guangdong Province in recent years, including fifty-one inbred lines, twelve restorer lines, six maintainer lines, and one P/TGMS line, were collected and planted for genotyping the rice bacterial blight resistance genes (Lu et al., 2019). The elite *indica* variety YueJinYinZhan (YJYZ) and the P/TGMS line YueJing1S (YJ1S) were used as recipient parents for disease resistance breeding in this study. XinHuangZhan (XHZ) and ‘V5400’, two *indica* type varieties harboring homozygous *Xa23* and conferring high broad-spectrum resistance to rice bacterial blight, were used as the donors of *Xa23*. On the other hand, ‘V5400’, harboring homozygous *Pi2* and conferring high resistance to rice blast, was used as the donor of *Pi2* for novel P/TGMS-line breeding. The breeding schemes for the novel inbred lines and P/TGMS lines are illustrated in **Figure 1**. All of the materials were planted using normal field management practices in an area of 16.7 × 16.7 cm at the experimental fields of Guangdong Academy of Agricultural Sciences (GDAAS), Guangzhou city, Guangdong Province, China.

### PCR-Based Genotyping and MAS Breeding

Plant genomic DNA was extracted from fresh leaves of the heading-stage plants using a previously described CTAB protocol (Liu et al., 1997). The molecular marker for genotyping *Xa4* was the co-dominant simple sequence repeat (SSR) marker RM224, that for *Xa7* was the sequence-tagged site (STS) marker STSP3, those for *Xa21* were the co-dominant STS marker pTA248 and the optimized functional marker U1/I2, those for *Xa23* were the SSR marker RM206 and the optimized marker P23, and that for *Xa27* was the co-dominant marker XA27-Co (Chunwongse et al., 1993; Chen et al., 2000; Pai et al., 2003; Porter et al., 2003; Sun et al., 2003; Gu et al., 2005; Zhou et al., 2011). The markers RM206 and P23 were used for MAS breeding to develop novel rice bacterial blight-resistant varieties with *Xa23*. In addition, the SSR marker RM527 and the Indel marker S29742 were used for genotyping the rice blast resistance gene *Pi2* and for MAS breeding (Deng Y. et al., 2006; Chen et al., 2008). Notably, MAS combining with phenotypic selection was used throughout the breeding process. PCR amplification was performed on a Bio-Rad C1000 Touch Thermal Cycler (USA) and the protocol for PCR amplification with the appropriate parameters was described previously (Wang et al., 2005). The PCR product for the marker RM206 was resolved on an 8% polyacrylamide gel, and other products were resolved on a 3.0% agarose gel in 1× TBE buffer. The primers used in this study are listed in **Table S3**.

### Evaluation of Rice Bacterial Blight Resistance

*Xoo* strains were grown on PSA medium at 28°C for 2–3 days, and the bacterial cells were suspended in sterile water at an optical density of 0.5 (OD600). 161BYZ-2 and 13WH-D, the pathotype IV and IX *Xoo* strains, respectively, were used for artificial inoculation with bacterial blight separately through the leaf-clipping method as described previously (Lu et al., 2019). All plants were inoculated at the heading stage. Photos of disease symptoms were taken two weeks after inoculation, and lesion length was measured three weeks after inoculation to evaluate the level of rice bacterial blight resistance. The evaluation standard of bacterial blight resistance was referred to previous reference (Liu et al., 2016).

### Fertility-Sterility Alteration Tested for the P/TGMS Lines

In 2016, the two P/TGMS lines, namely, Tai S and Wo S, were sown every 10 days from February 22^nd^ to March 23^rd^ in the early cropping season and from July 21^st^ to August 20^th^ in the later cropping season, respectively, in the experimental fields of GDAAS, Guangzhou city, Guangdong Province, China. YJ1S was used as the control. The dynamic pollen fertility of these P/TGMS lines was observed at several-day intervals from May 18^th^ to June 11^th^ during the early cropping season and September 25^th^ to October 27^th^ during the later cropping season in 2016. Male fertility conversion and multiplication of these P/TGMS lines were conducted using an artificial intelligence-based cold-water serial irrigation system. The cold water (20–22°C) irrigation treatment was started at the phase of differentiation of the secondary rachis branch and spikelet primordia (young panicle differentiation phase III) and was performed until the meiotic division of the pollen mother cell (phase VI).

### Production Measurement and Statistical Analysis

Hybrid combinations were made through several cytoplasm male sterility (CMS) and P/TGMS lines crossed with the restorer lines, and the F1 hybrids were grown in the field in an area of 16.7 × 16.7 cm using normal field management practices. At maturity, all of the plants in each plot were harvested, and the grain yield of the hybrid combinations was measured according to a method described previously (Wang S. et al., 2018). Mean phenotypic values were compared using Student’s *t* test and the data were analyzed using Microsoft Excel 2013 as previously described (Wang S. et al., 2018).

## Results

### Identification of Bacterial Blight Resistance Genes in the Tested Cultivars

Understanding the distribution of disease resistance genes in rice cultivars is beneficial not only for rice breeding but also for the rational distribution of cultivars. In the present study, we collected seventy main cultivars from Guangdong Province, in the southern China rice region, and identified the distribution of the bacterial blight resistance genes using the corresponding specific markers, including *Xa4*, *Xa7*, *Xa21*, *Xa23*, and *Xa27*, which act in a dominant manner with broad-spectrum resistance against BB and have been widely utilized in rice resistance breeding. The detailed identification results are shown in **Table S1** and **Figure S1**.

The results showed that 61 cultivars carried *Xa4*, accounting for 87.1%; and three cultivars, namely, Lvhuangzhan (LHZ), Qiu B, and Yuefeng B, carried *Xa27*, accounting for only 4.3% (**Table S1**). However, none of the tested cultivars carried *Xa7*, *Xa21*, or *Xa23*. On the other hand, 33 cultivars had moderate resistance or more to pathotype IV *Xoo* strains, and only two cultivars, namely, Huanglizhan (HLZ) and LHZ had moderate resistance to pathotype IX *Xoo* strains (**Table S1**). These results indicated that *Xa4* had been widely used in rice breeding for BB resistance in Guangdong but was unable to meet the current requirements for rice disease resistance breeding. Two cultivars, HLZ and LHZ, have potential as excellent resistant germplasms for rice disease resistance breeding. The disease resistance mechanism of HLZ and LHZ should be further investigated to facilitate the subsequent marker-assisted selection.

### Development of Elite Rice Varieties and Novel P/TGMS Lines

**Figure 2:**
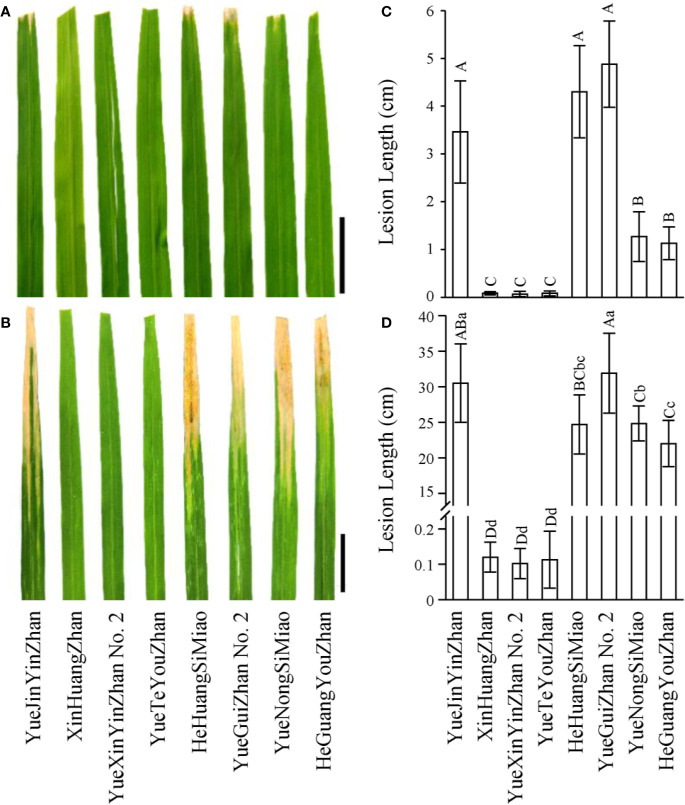
Disease reaction patterns of rice varieties to *Xoo* strains. **(A, B)** Leaves of rice varieties are presented to show the lesion patterns for the pathotype IV and IX *Xoo* strains, respectively. Pictures were taken on the 14^th^ day after inoculation. Scale bars: 5 cm. **(C, D)** Statistical analysis of the lesion lengths of rice varieties inoculated with the pathotype IV and IX *Xoo* strains, respectively. Lesion length was measured on the 21^st^ day after inoculation. Data are given as the means with SDs (n = 10). The uppercase and lowercase letters above the bars represent significant difference levels P < 0.01 and P < 0.05, respectively.

**Figure 3:**
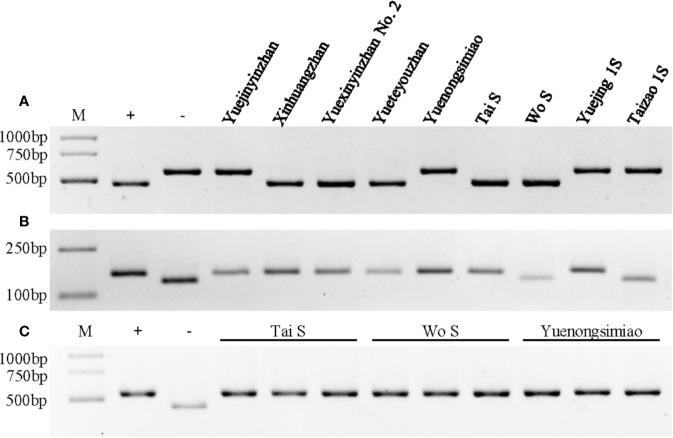
Genotype identification of *Xa23*, *Xa4*, and *Pi2* in different parental lines. **(A)** Genotype identification of the *Xa23* gene using the optimized marker P23. The positive (+) and negative control (−) were CBB23 and JG30, respectively. **(B)** Genotype identification of the *Xa4* gene using the co-dominant SSR marker RM224. The positive (+) and negative control (−) were IR64 and IR24, respectively. **(C)** Genotype identification of the *Pi2* gene using the Indel marker S29742. The positive (+) and negative control (−) were C101A51 and Lijiangxintuanheigu (LTH), respectively. M represents the DNA marker.

Based on the distribution of bacterial blight resistance genes and phenotypic results, it is necessary to explore disease-resistant germplasms or have excellent gene resources with broad-spectrum and high resistance to bacterial blight to improve or breed new varieties. First, we observed that XHZ, carrying the broad-spectrum and durable resistance gene *Xa23* derived from CBB23, exhibited a typical hypersensitive disease resistance response to pathotype IV and also to pathotype IX *Xoo* strains (**Figures 2** and **3A**), which was consistent with the results of previous studies (Luo et al., 2013; Wang et al., 2015; Zeng L. et al., 2017). Then, we used XHZ as an important resistant donor for disease resistance improvement. As shown in the schematic (**Figure 1A**), XHZ harboring homozygous *Xa23* was crossed with YJYZ, an excellent rice variety with high yield and high sensitivity to pathotype IX *Xoo* strains (**Figures 2B, D**). We used MAS combined with phenotypic selection for pedigree breeding and filtered out the high-quality and high resistance line 823^#^ with homozygous *Xa23* gene from the F5 generation in later cropping season of 2013. Finally, we bred a new excellent rice variety with high yield and high quality called YueXinYinZhan No.2 (YXYZ2, **Figure 1A**) from line 823^#^, and this variety was authorized in 2018 (Guangdong authorized variety no. 20180041). Subsequently, we crossed the line 823^#^ with the large-panicle and high-yield intermediate material line 858^#^, which was derived from the high-quality and main cultivated rice variety YueNongSiMiao (YNSM). Then, we bred a new variety YueTeYouZhan (YTYZ) harboring homozygous *Xa23* gene in later cropping season of 2016 (**Figures 1A** and **3A**).

Previous results showed that *Xa23* confers dominant bacterial blight resistance throughout the growth period and plays an important role in disease resistance breeding of hybrid rice (Wang et al., 2015). In this study, the multi-resistant germplasm “V5400”, harboring homozygous *Xa23* and *Pi2* genes, as the donor of resistance genes was used for backcrossing with the recipient parent YJ1S, a high-quality pollen-free P/TGMS line with high combination ability (**Figure 1B**). In the BC2F6 generation in early cropping season of 2015, we picked out two lines, by16^#^ and by22^#^, showing excellent comprehensive agronomic traits and good outcrossing (**Figure 1B**). Genotyping analysis of resistance genes indicated that the two P/TGMS lines, tentatively named Tai S and Wo S, respectively, carried homozygous *Xa23* and *Pi2* (**Figures 3A, C**). Tai S exhibited similar heading date as YJ1S, while Wo S exhibited heading date approximately ten days later. In the field conditions, Tai S and Wo S exhibited complete male sterility until late October or early November, respectively, but exhibited conversion to male fertility and self-propagation under serial irrigation with cold water at temperatures below 23°C. These results indicated that the novel P/TGMS lines Tai S and Wo S had the characteristics of low critical sterility-inducing temperature and stable male sterility. Finally, Tai S and Wo S were released in 2018, and MAS combined with phenotypic selection was performed throughout the breeding process.

### The Novel Varieties and P/TGMS Lines Exhibited High Resistance to Rice Bacterial Blight

To test whether YXYZ2 and YTYZ show improved the bacterial blight resistance, the pathotype IV and IX *Xoo* strains were used for inoculation. The results showed that YJYZ and YNSM exhibited moderate resistance and resistance to the pathotype IV *Xoo* strain with lesion lengths 3.46 ± 1.07 cm and 1.27 ± 0.52 cm, respectively (**Figures 2A, C**), high susceptibility to the pathotype IX *Xoo* strain with lesion lengths more than 24 cm (**Figures 2B, D**). Notably, the bacterial blight resistance of YXYZ2 and YTYZ to pathotype IV and IX *Xoo* strains was significantly higher than that of YJYZ and YNSM, and these varieties exhibited a typical hypersensitive disease resistance response with lesion lengths less than 0.2 cm as XHZ (**Figure 2**). Similarly, we used the pathotype IV and IX *Xoo* strains for inoculation to evaluate the bacterial blight resistance of the two novel P/TGMS lines (**Figure S2**). The results showed that Tai S and Wo S were highly resistant to the pathotype IV and IX *Xoo* strains, with lesion lengths ranging from 0.10 to 0.28 cm (**Figure S2**). However, the other widely applied sterile lines for two- or three-line hybrid rice breeding in China were susceptible to the pathotype IV *Xoo* strains with lesion lengths ranging from 5.39 to 23.80 cm (**Figures S2A, C**) and exhibited high susceptibility to the pathotype IX *Xoo* strains with lesion lengths more than 23.5 cm (**Figures S2B, D**). Meanwhile, we investigated the status of *Xa4* in those improved lines and their parental lines (**Figure 3B**). The results showed that *Xa4* was present in all of the parental lines and improved lines except Wo S, which indicated that the improved lines, YXYZ2, YTYZ, and Tai S, pyramided *Xa23* and *Xa4* at least (**Figures 3A, B**). The above results indicated that the broad-spectrum resistance gene *Xa23* can be effectively applied to rice disease resistance breeding. In addition, the two P/TGMS lines Tai S and Wo S, which carried the homozygous *Pi2* gene (**Figure 3C**), were resistant to rice blast, as determined by field resistance identification in a natural blast nursery for many years (**Table S2**). These results suggested that MAS coupled with excellent gene pyramiding is helpful for improving target traits rapidly and accelerating the breeding process.

### Evaluation of Hybrids Derived From the Improved P/TGMS Lines or Cultivars

The development of hybrid rice has greatly contributed to global food security and continues to provide a fundamental guarantee for food supply (Yuan, 2017). In the field conditions, Tai S and Wo S exhibited complete male sterility, and the period of male sterility for the two P/TGMS lines was more than 150 days, as determined by sowing in stages and performing fertility identification in consecutive years. Furthermore, Tai S and Wo S exhibited high fertility restoration efficiency for multiplication under cold-water serial irrigation. Thus, we bred several hybrid combinations derived from Tai S and Wo S, namely, Tai S/YueYaSiMiao (YYSM) and Wo S/YYSM, Tai S/YueGuiZhan No.2 (YGZ2) and Wo S/YGZ2; these lines were planted, and the yields were evaluated in later cropping season of 2018 and early cropping season of 2019, respectively. The results showed that the yields of the above hybrids were significantly higher than that of the control variety Guangbayou 2168 (GBY2168) or Shenliangyou 58 xiangyouzhan (SLY58XYZ), increasing by 4.8–20.1% (**Figure S5B**). These results indicated that the two novel P/TGMS lines Tai S and Wo S are suitable for practical breeding of two-line hybrid rice.

**Figure 4:**
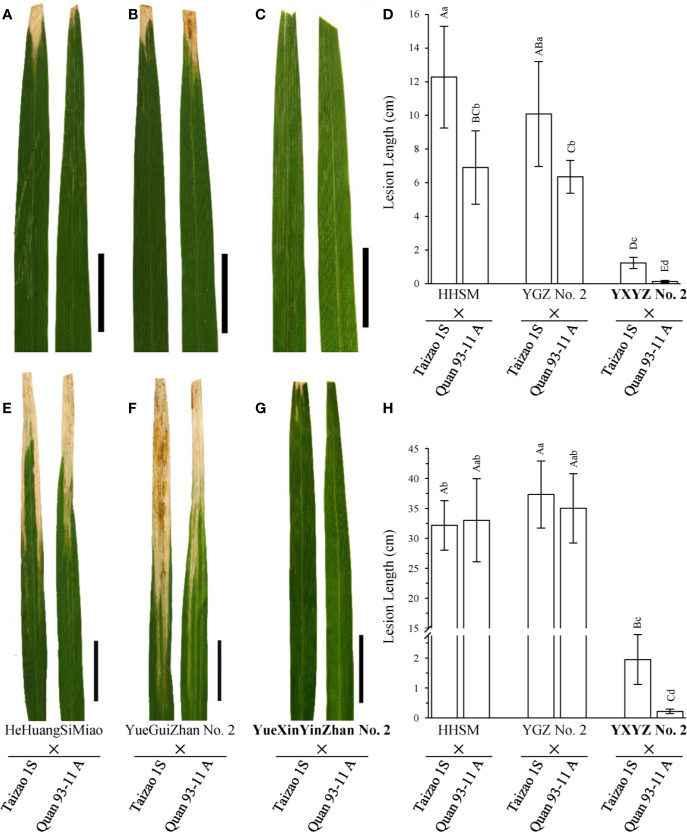
Comparison of the disease response of hybrid combinations of novel rice varieties. **(A–C**, **E–G)** Leaves of hybrids are presented to show the lesion patterns to the pathotype IV and IX *Xoo* strains, respectively. Pictures were taken on the 14^th^ day after inoculation. Scale bars: 5 cm. **(D, H)** Statistical analysis of the lesion lengths of hybrids inoculated with the pathotype IV and IX *Xoo* strains, respectively. Lesion length was measured on the 21^st^ day after inoculation. Data are given as the means with SDs (n = 10). The uppercase and lowercase letters above the bars represent significant difference levels P < 0.01 and P < 0.05, respectively.

Subsequently, we used two elite restorer lines, namely, HeHuangSiMiao (HHSM) and YGZ2, for hybridization with a series of P/TGMS lines and evaluated the resistance response of the derived hybrid combinations to bacterial blight (**Figure S3**). HHSM and YGZ2 exhibited moderate resistance to the pathotype IV *Xoo* strains with lesion lengths less than 5 cm (**Figures 2A, C**) and high susceptibility to the pathotype IX *Xoo* strains with lesion lengths more than 24 cm (**Figures 2B, D**). Compared with the results for hybrid combinations derived from other P/TGMS lines exhibiting susceptibility or high susceptibility to bacterial blight, the hybrid combinations derived from Tai S and Wo S showed a high-resistance or resistance response to bacterial blight, with lesion lengths ranging from 0.35 to 2.69 cm (**Figure S3**). Remarkably, the novel cultivars YXYZ2 and YTYZ are not only being excellent conventional rice varieties with high grain yield but also being elite restorer lines (**Figure S5**). The yield of the hybrid combinations, Tai S/YXYZ2 and Fa S/YTYZ, reached more than 9.4 t/ha in the trial conducted in early cropping season of 2018, which was significantly higher than that of the control variety Tianyou 3618 (TY3618), with an increase of more than 15% (**Figure S5B**). Next, we also evaluated the bacterial blight resistance of the hybrid combinations derived from YXYZ2 and YTYZ (**Figure 4** and **Figure S4**). The hybrid combinations derived from YXYZ2 and YTYZ exhibited enhanced bacterial blight resistance compared with the other hybrids derived from the same sterile lines (**Figure 4** and **Figure S4**). Bacterial lesion lengths of these hybrids ranged from 0.07 to 0.41 cm with the exception of the combination of Taizao 1S with YXYZ2, which were more than 1.2 cm (**Figure 4** and **Figure S4**). These results indicated that the dominant resistance gene *Xa23* could improve the bacterial blight resistance of hybrid and inbred rice, and the resistance of hybrid rice would probably be affected by the genetic background in the application of *Xa23*. Meanwhile, the results showed that the hybrids derived from Taizao 1S, without harboring *Xa4* (**Figure 3B**), were more susceptible to the pathotype IV strains than those from Quan 93-11A (**Figures 4A–D**). Similarly, the hybrids derived from Shen 08S were significantly susceptible to the pathotype IV strains compared to those from Fulong S2 or Li 1S (**Figures S3E, F**).

## Discussion

Rice plays a predominant role in the major strategies for demand-oriented national food security and achieving a guaranteed high and stable yield of rice is a fundamental goal (Zeng D. et al., 2017). However, for rice production and security, the major obstacle caused by multiple pathogens should be addressed (Ke et al., 2017). It is widely accepted that the utilization of broad-spectrum and durable resistance for rice improvement is the most cost-effective approach to preventing the loss of rice yield caused by various diseases (Khan et al., 2014; Li et al., 2017). Although an increasing number of bacterial blight resistance (*R*) genes have been identified and cloned in past decades, and main cultivars also showed resistant improvement *to Xoo strains* through the application of *R* genes (Vikal and Bhatia, 2017; Chukwu et al., 2019), with the continuous emergence of new strong pathogenic *Xoo* strains, the resistance of the main cultivars is at risk of loss.

In the present study, we genotyped and detected the distribution of a set of *R* genes, *i.e. Xa4*, *Xa7*, *Xa21*, *Xa23*, and *Xa27*, in seventy main cultivars from Guangdong Province using the corresponding specific markers. Among these detected genes, our results indicated that *Xa4* was probably the most widely utilized *R* gene in rice breeding for BB resistance in Guangdong (**Table S1**). Meanwhile, it is worth noting that the maintainers, such as Guang 8B, Wufeng B, and Tianfeng B, corresponding to the widely applied CMS lines in rice hybrids harbored *Xa4*, which mediated a race-specific and durable resistance against *Xoo* at all stages of rice growth with dominant inheritance (Sun et al., 2003; Hu et al., 2017). These results indicated that *Xa4* had also been widely utilized in rice hybrid breeding programs, which was consistent with the previous studies (Zhang et al., 2002; Sun et al., 2003; Hu et al., 2017). However, the resistance of *Xa4* had weakened and even been overcome by the more virulent BB strains (Zhang et al., 2002; Chen et al., 2011). On the other hand, the strong virulent BB strains of pathotype V *Xoo* had replaced pathotype IV *Xoo* strains, and pathotype V had become the preponderant pathotype in South China; meanwhile, the new and the most virulent pathotype, pathotype IX, evolved quickly (Zeng et al., 2005; Chen et al., 2018). In the present study, our results showed that only two cultivars had moderate resistance to the strongly virulent pathotype IX *Xoo* strains (**Table S1**), which indicated that *Xa4* has been unable to improve BB resistance in rice disease resistance breeding in Guangdong. Consequently, the exploration and utilization of the novel resistance resources play a pivotal role in rice resistance breeding for protection against the rapidly evolving *Xoo* races.

Bacterial blight races continue to evolve with artificial or natural selection and the utilization of BB resistance genes (Chukwu et al., 2019). As a result, the most virulent *Xoo* strains develop rapidly, and the preponderant *Xoo* pathotypes are also being frequently replaced (Zeng et al., 2005; Chen et al., 2018). Previous studies have indicated that *Xa23*, a transcription activator-like (TAL) effector dependent *R* gene, confers broad-spectrum resistance and provides strong resistance to all-natural *Xoo* strains tested so far at all growth stages of rice (Wang et al., 2015). Notably, *Xa23* can trigger a strong hypersensitive response in crop plants (Vikal and Bhatia, 2017), and cultivars such as CBB23 and XHZ carrying the *Xa23* gene exhibited high (grade 1) resistance to strains IV and V of *Xoo* from South China (Luo et al., 2013; Zeng L. et al., 2017). In the present study, our results showed that XHZ exhibited a typical hypersensitive disease resistance response to strain IV and also to strain IX of *Xoo* (**Figure 2**). Subsequently, we developed a series of highly disease-resistant intermediate materials and successfully bred two excellent rice varieties, namely, YXYZ2 and YTYZ, using XHZ as a resistant resource through MAS combined with phenotypic selection (**Figure 1A**). As expected, the two excellent varieties YXYZ2 and YTYZ exhibited typical hypersensitive disease resistance to the virulent *Xoo* pathotype IV or IX with high and stable grain production (**Figure 2** and **Figure S5A**). In addition, the parental lines and derived hybrid combinations without harboring *Xa23* resistance gene exhibited different responses to the pathotype IV *Xoo* strains and high susceptibility to the pathotype IX *Xoo* strains (**Figures 2** and **4**, **Figures S3** and **S4**). These results indicated that the resistance conferred against pathotype IV *Xoo* strains was statistically significant, although this strain appears less aggressive.

Since the 1970s, the exploitation and utilization of heterosis in hybrid rice had led to a second leap in rice grain yield and to significant achievements (Qian et al., 2016; Yuan, 2017). However, breeding of disease-resistant and high-quality male sterile lines is a strategic problem that has long hindered the development and utilization of hybrid rice. Currently, the development of rice functional genomics is greatly accelerating the rational design and molecular breeding to enable new breakthroughs in rice production (Qian et al., 2016; Zeng D. et al., 2017). Nevertheless, improving and enhancing disease resistance is important for guaranteeing a high and stable yield of rice. Previous studies indicated that the rice genetic background would influence the *R*-gene-mediated resistance to *Xoo* in rice and, thus, hinder the utilization of a single *R* gene for disease resistance to BB in hybrid rice due to the heterozygous genetic background or incomplete dominance (Luo et al., 2012; Zeng L. et al., 2017). For recessive resistance genes, non-transgenic resistant varieties can be obtained quickly by gene editing, but these genes cannot be directly utilized in hybrid rice breeding (Kim et al., 2019; Li C. et al., 2020). Notably, *Xa23* confers complete dominant bacterial blight resistance independently of genetic background during the whole growth period; thus, this gene has been widely adopted in rice breeding programs, and several *Xa23*-containing hybrid rice varieties have been released (Wang et al., 2014; Wang et al., 2015). On the other hand, the application of P/TGMS lines has effectively broadened the genetic diversity in sterile line breeding and is a breakthrough in the breeding of disease-resistant and high-quality male sterile lines (Ma and Yuan, 2015). Here, we bred two novel P/TGMS lines, namely, Tai S and Wo S, harboring homozygous *Xa23* through backcrossing with MAS breeding (**Figures 1B** and **3A**), and the derived hybrid combinations exhibited high resistance against the pathotype IV and IX *Xoo* strains as the two female parents (**Figures S2** and **S3**). In addition, the hybrid combinations using YXYZ2 and YTYZ as restorer lines also exhibited high resistance to bacterial blight with high yield (**Figure 4**, **Figures S4** and **S5**). These results indicated that *Xa23* has wide application value due to its high and broad-spectrum resistance with complete dominant inheritance in inbred and hybrid rice resistance breeding. Remarkably, the *Xa23* gene mediated resistance to *Xoo* would be probably influenced by the genetic background in hybrids (**Figure 4** and **Figure S3**), indicating that *Xa23* coupled with other resistance genes needs to be taken into consideration in future applications.

As mentioned above, we successfully bred two excellent cultivars and two novel P/TGMS lines harboring homozygous *Xa23* and exhibiting high resistance against BB through conventional breeding aided by MAS. However, the *Xoo* or pathotypes keep evolving and overcoming the barrier presented by the *R* genes. Meanwhile, continuous planting of cultivars with a single major *R* gene imposes a strong selection on pathogen evolution and variation and results in resistance breakdown (Kim, 2018; Chukwu et al., 2019). Although the *Xa23* locus confers high resistance to virtually all the *Xoo* races tested, it is possible that the *Xa23*-containing rice varieties would lose BB resistance if widely distributed and subjected to long-term planting and application (Wang et al., 2014; Wang et al., 2015). In other words, the vertical resistance controlled by a single resistance gene would be lost due to the arms race between plants and pathogens, as described by the Red Queen’s hypothesis. Fortunately, an increasing number of BB *R* genes that confer resistance against different *Xoo* strains *via* multiple mechanisms that have been identified and cloned (Chukwu et al., 2019). Recently, Xu et al. (2019) and Oliva et al. (2019) created new germplasms with robust and broad-spectrum resistance to BB using CRISPR/Cas9-mediated genome editing to introduce mutations in three effector-triggered susceptibility-associated *SWEET* genes, namely, *Xa13*/*OsSWEET11*, *Xa25*/*OsSWEET13*, and *Xa41*/*OsSWEET14*, successively. Therefore, pyramiding of multiple *R* genes is one of the more conducive and effective approaches to enhancing the horizontal resistance of rice varieties and achieving broad-spectrum and durable resistance (Luo et al., 2012; Suh et al., 2013; Pradhan et al., 2015; Luo et al., 2016; Oliva et al., 2019; Xu et al., 2019). In the present study, the improved lines, YXYZ2, YTYZ, and Tai S, pyramided *Xa23* and *Xa4* at least (**Figures 3A, B**), which could have been better than the *Xa23* alone for disease resistance and durability, although *Xa4* alone was unable to meet the current requirements for rice disease resistance breeding. Notably, pyramiding of multiple *R* genes in rice should not be accompanied by yield penalties or drawbacks for other traits (Antony et al., 2010; Li et al., 2012; Xu et al., 2019). In general, the exploitation of the new resistance resources and the molecular identification of novel genes for disease resistance are the key components for the rational design of rice resistance breeding.

## Data Availability Statement

The raw data supporting the conclusions of this article will be made available by the authors, without undue reservation.

## Author Contributions

SW and XH designed the experiments and wrote the manuscript. SW, JX, and XH edited the manuscript. SW, WL, ZL, and DL performed the experiments. SW and XW analyzed the data. All authors contributed to the article and approved the submitted version.

## Funding

This work was supported by grants from the National Key R&D Program of China (2017YFD0100102), The National Natural Science Foundation of China (31471175), Science and Technology Programme of Guangdong Province, China (2015A020209043, 2017A070702006), Modern Agricultural Industry Technology System of Guangdong Province, China (2019KJ105), Joint Research on High Quality Rice Varieties (Yuecainong [2019]73), and the Special Fund for Science and Technology Innovation Strategy (Construction of High-level Academy of Agricultural Sciences) (Foundation of President of Guangdong Academy of Agricultural Sciences in China, 201924).

## Conflict of Interest

The authors declare that the research was conducted in the absence of any commercial or financial relationships that could be construed as a potential conflict of interest.
