# Supplementary material for: Distribution of Bacterial Blight Resistance Genes in the Main Cultivars and Application of Xa23 in Rice Breeding
Source: Front Plant Sci. 2020 Aug 31;11:555228. doi: 10.3389/fpls.2020.555228 (PMC7488846; doi:10.3389/fpls.2020.555228)
Supplement: Supplementary file 1 [file DataSheet_1.docx]

**Supporting Informations**

**Figure S1. Genotypic identification of the bacterial blight resistance genes in the main cultivars.** (A) Genotypic identification of the *Xa4* gene using the SSR marker RM224. The positive (+) and negative control (-) were IR64 and IR24, respectively. (B) Genotypic identification of the *Xa7* gene using the sequence-tagged site marker STSP3. The positive (+) and negative control (-) were IRBB7 and LTH, respectively. (C) Genotypic identification of the *Xa21* gene using the co-dominant marker pTA248. The positive (+) and negative control (-) were IRBB21 and LTH, respectively. (D) Genotypic identification of the *Xa23* gene using the optimized marker P23. The positive (+) and negative control (-) were CBB23 and JG30, respectively. (E) Genotypic identification of the *Xa27* gene using the co-dominant marker XA27-Co. The positive (+) and negative control (-) were IRBB27 and LTH, respectively. M represents DNA marker.

**Figure S2. Disease response patterns of rice male sterile lines against *Xoo* strains.** (A and B) Leaves of rice male sterile lines were presented to show the lesion patterns to the pathotype IV and IX *Xoo* strains, respectively. Pictures were taken on the 14^th^ day after inoculation. Scale bars: 5 cm. (C and D) Statistical analysis of the lesion lengths of rice male sterile lines inoculated with the pathotype IV and IX *Xoo* strain, respectively. Lesion length was measured on the 21^st^ day after inoculation. Data are given as the means with SDs (n = 10). The uppercase and lowercase letters above the bars represent significant difference levels P < 0.01 and P < 0.05, respectively.

**Figure S3. Comparison of the disease response of hybrid combinations of newly bred P/TGMS lines.** (A and C) Leaves of hybrids were presented to show the lesion patterns to the pathotype IV *Xoo* strain. Scale bars: 5 cm. (B and D) Leaves of hybrids were presented to show the lesion patterns to the pathotype IX *Xoo* strain. Scale bars: 5 cm. Pictures were taken on the 14^th^ day after inoculation. (E and F) Statistical analysis of the lesion lengths of hybrids derived from Hehuangsimiao and Yueguizhan No.2, respectively. Lesion length was measured on the 21^st^ day after inoculation. Data are given as the means with SDs (n = 10). The uppercase and lowercase letters above the bars represent significant difference levels P < 0.01 and P < 0.05, respectively.

**Figure S4. Comparison of the disease response of hybrid combinations of novel rice varieties.** (A-C and E-G) Leaves of hybrids were presented to show the lesion patterns to the pathotype IV and IX *Xoo* strains, respectively. Pictures were taken on the 14^th^ day after inoculation. Scale bars: 5 cm. (D and H) Statistical analysis of the lesion lengths of hybrids inoculated with the pathotype IV and IX *Xoo* strain, respectively. Lesion length was measured on the 21^st^ day after inoculation. Data are given as the means with SDs (n = 10). The uppercase and lowercase letters above the bars represent significant difference levels P < 0.01 and P < 0.05, respectively.

**Figure S5. Yield evaluation of the novel rice varieties and hybrid combinations.** The uppercase and lowercase letters above the bars represent significant difference levels P < 0.01 and P < 0.05, respectively.


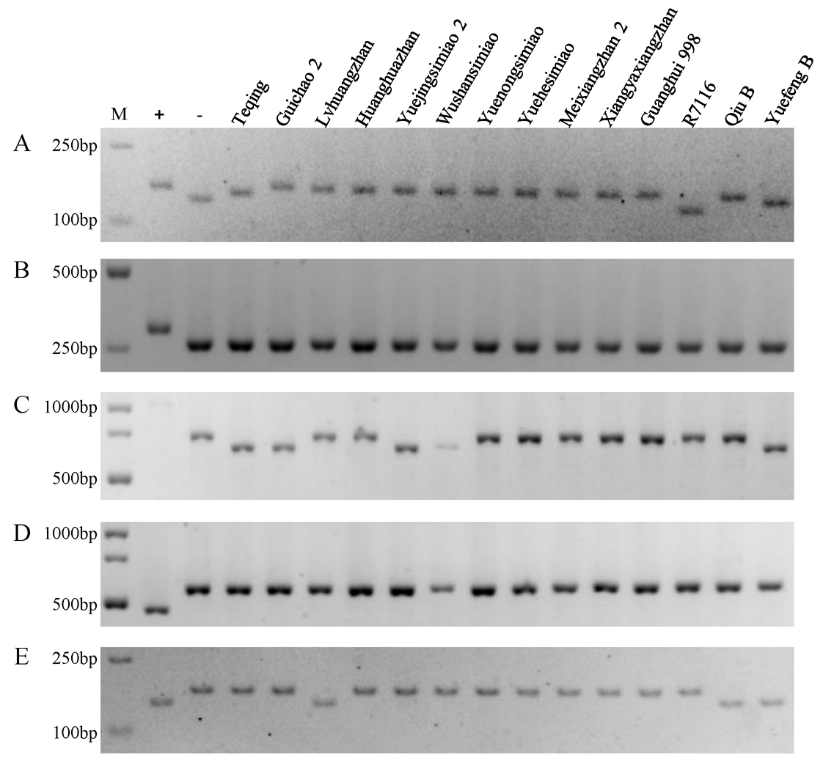


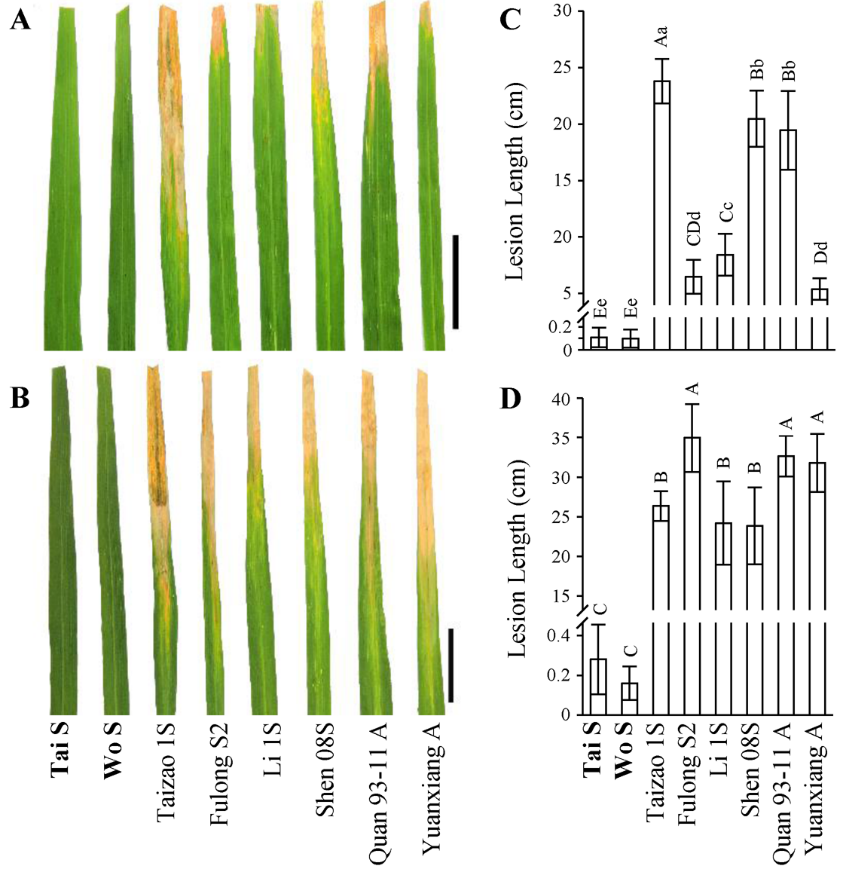


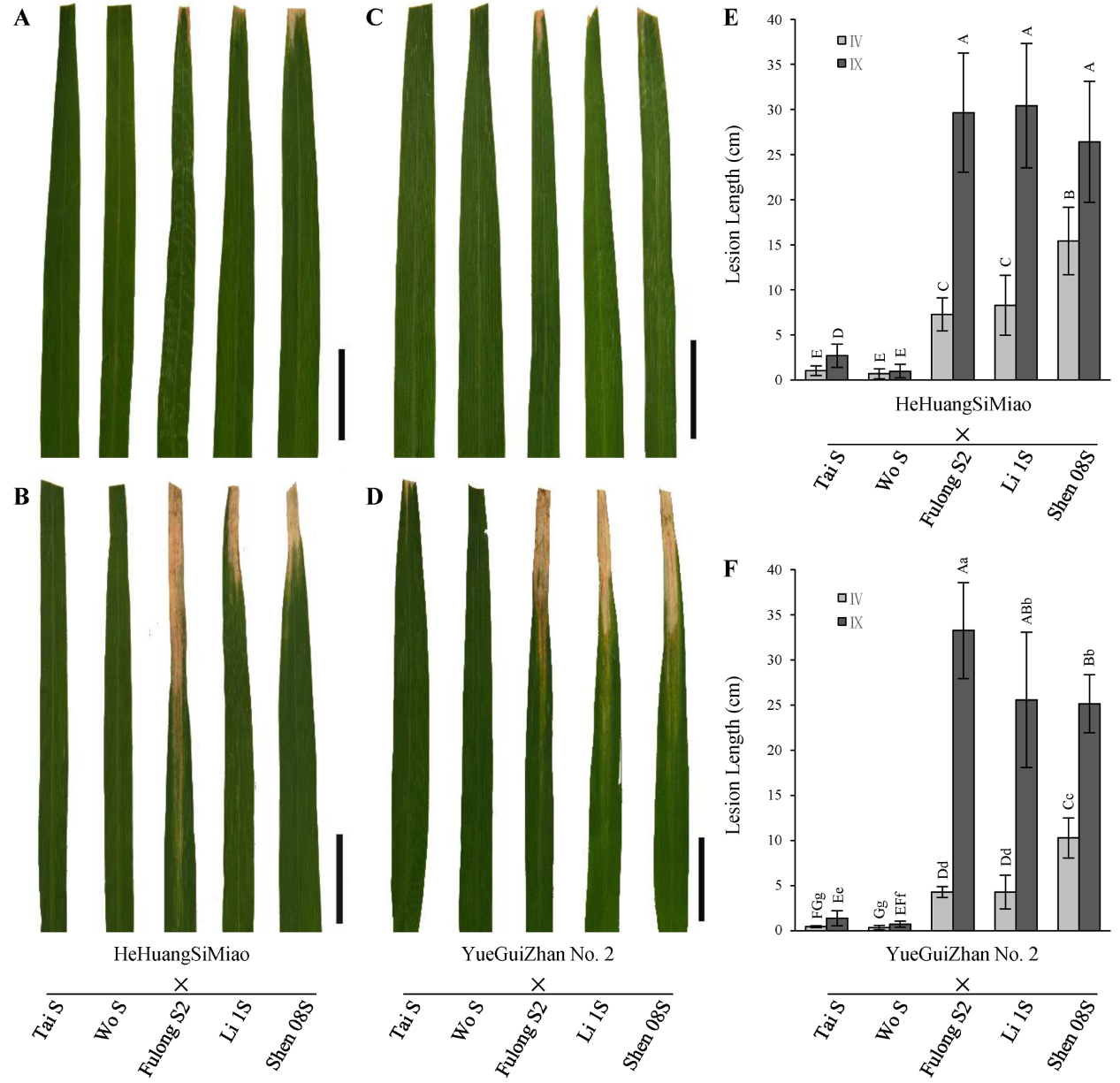


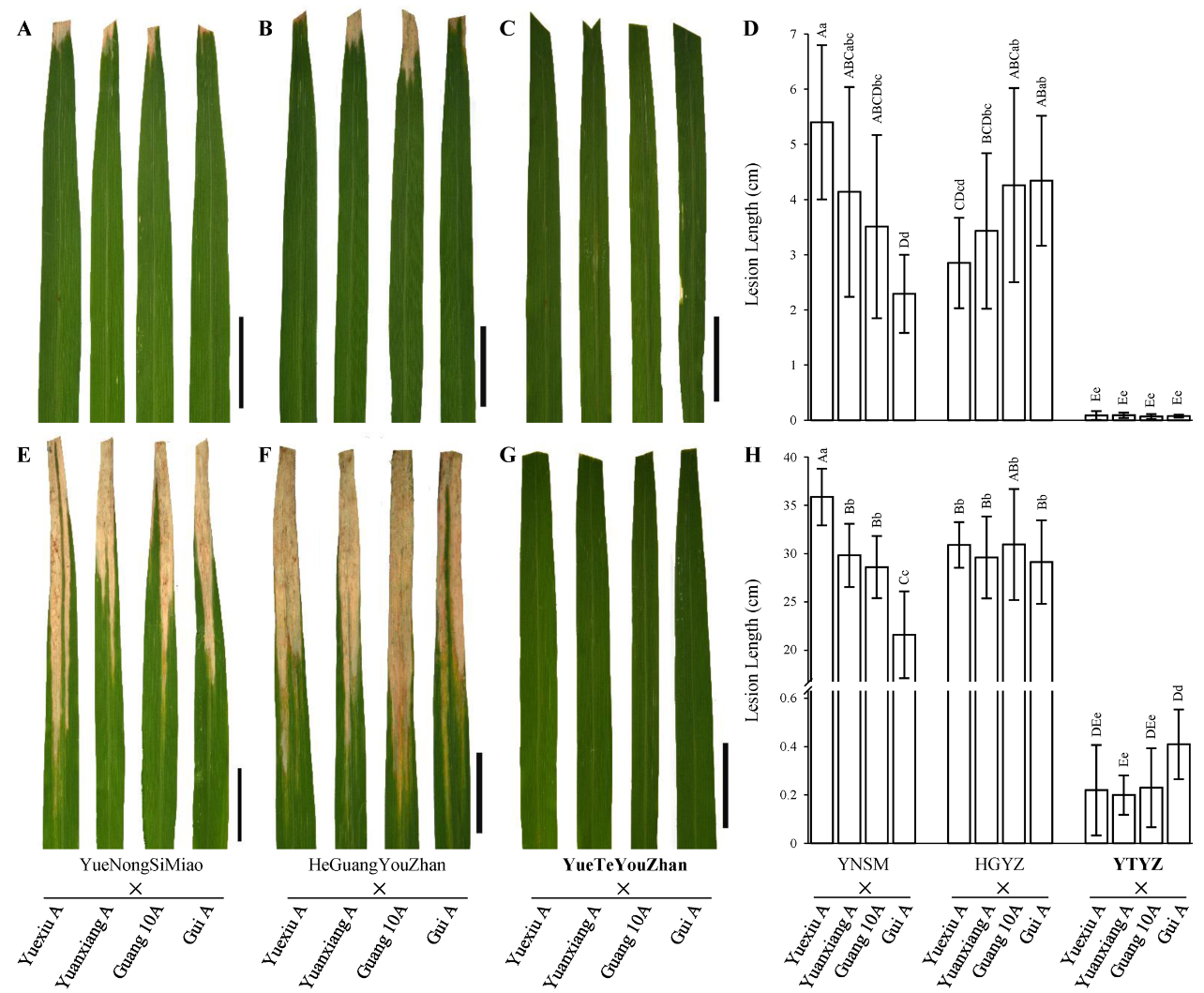


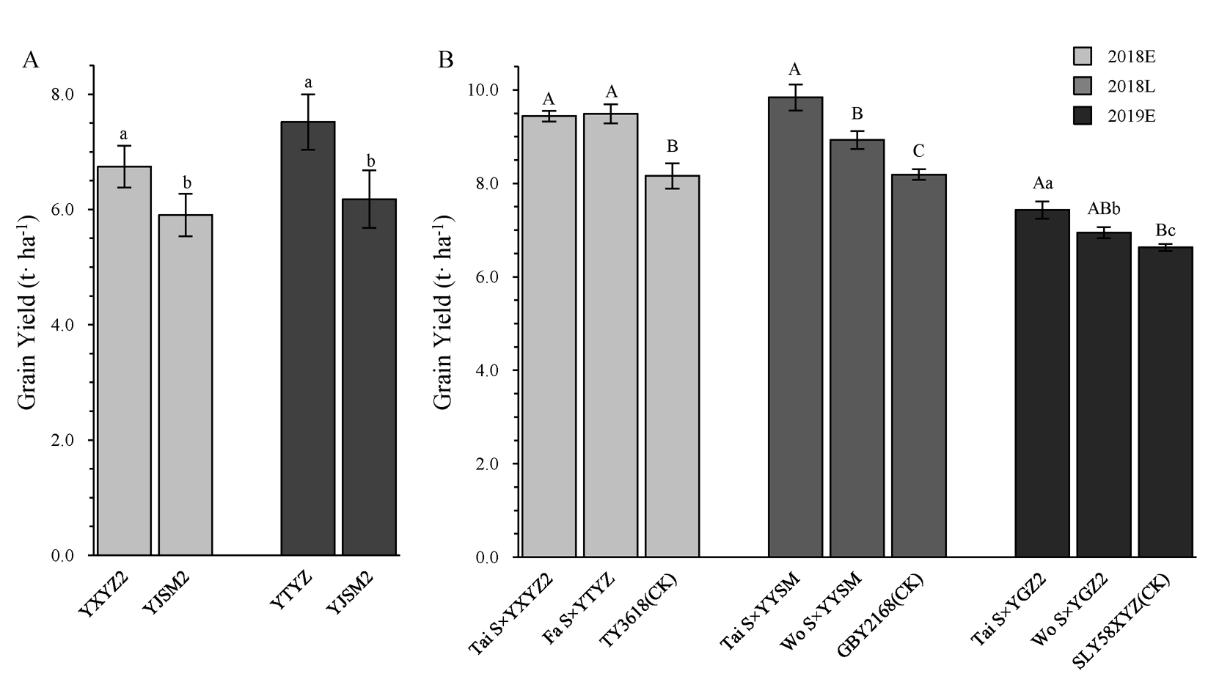


**Supplemental Tables:**

Table S1. Summary of the main rice cultivars carrying R genes and resistance analysis with the bacterial blight pathotype IV and IX strain.

Table S2. Resistance level of the novel P/TGMS lines against rice blast in the natural blast nursery.

Table S3. Primers used in this study.

Table S1. Summary of the main rice cultivars carrying R genes and resistance analysis with the bacterial blight pathotype IV and IX strain.

| **No.** | **Materials** | ***Xa4*** | ***Xa7*** | ***Xa21*** | | ***Xa23*** | | ***Xa27*** | **Resistance level** | |
| --- | --- | --- | --- | --- | --- | --- | --- | --- | --- | --- |
|  |  | **RM224** | **STS P3** | **pTA248** | **U1/I2** | **RM206** | **P23** | **Xa27-Co** | **IV** | **IX** |
| 1 | Yuejingsimiao2 | + | - | - | - | - | - | - | 3 | 7 |
| 2 | Yuefengzhan | + | - | - | - | - | - | - | 3 | 5 |
| 3 | Zhong'erruanzhan | + | - | - | - | - | - | - | 3 | 5 |
| 4 | Nanfengnuo | + | - | - | - | - | - | - | 5 | 9 |
| 5 | Yuexiangzhan | + | - | - | - | - | - | - | 3 | 5 |
| 6 | Qishanzhan | + | - | - | - | - | - | - | 5 | 7 |
| 7 | Fenghuazhan | + | - | - | - | - | - | - | 3 | 7 |
| 8 | Qilisimiao | + | - | - | - | - | - | - | 3 | 7 |
| 9 | Huanghuazhan | + | - | - | - | - | - | - | 5 | 9 |
| 10 | Yuxiangyouzhan | + | - | - | - | - | - | - | 3 | 7 |
| 11 | Yinjingruanzhan | + | - | - | - | - | - | - | 3 | 7 |
| 12 | Huanglizhan | + | - | - | - | - | - | - | 3 | 3 |
| 13 | Meixiangzhan2 | + | - | - | - | - | - | - | 5 | 9 |
| 14 | Teqing | - | - | - | - | - | - | - | 3 | 7 |
| 15 | Yexianzhan8 | + | - | - | - | - | - | - | 3 | 7 |
| 16 | Yuexiuzhan | + | - | - | - | - | - | - | 3 | 7 |
| 17 | Huangsizhan | + | - | - | - | - | - | - | 3 | 7 |
| 18 | Yesizhan | + | - | - | - | - | - | - | 3 | 7 |
| 19 | Guangyuan5 | + | - | - | - | - | - | - | 3 | 7 |
| 20 | Guinongzhan | + | - | - | - | - | - | - | 5 | 7 |
| 21 | Wushansimiao | + | - | - | - | - | - | - | 3 | 7 |
| 22 | Hefengzhan | + | - | - | - | - | - | - | 3 | 7 |
| 23 | Yuenongsimiao | + | - | - | - | - | - | - | 3 | 7 |
| 24 | Mabayinzhan | + | - | - | - | - | - | - | 3 | 7 |
| 25 | Huangguangyouzhan | + | - | - | - | - | - | - | 3 | 7 |
| 26 | Jinnongsimiao | + | - | - | - | - | - | - | 3 | 7 |
| 27 | Hefengsimiao | + | - | - | - | - | - | - | 3 | 7 |
| 28 | Yuehesimiao | + | - | - | - | - | - | - | 3 | 7 |
| 29 | Huahang31 | + | - | - | - | - | - | - | 3 | 9 |
| 30 | Xiangyaxiangzhan | + | - | - | - | - | - | - | 3 | 7 |
| 31 | Hemeizhan | + | - | - | - | - | - | - | 3 | 7 |
| 32 | Fengmeizhan | + | - | - | - | - | - | - | 3 | 5 |
| 33 | Huahangsimiao | - | - | - | - | - | - | - | 9 | 9 |
| 34 | Texianzhan25 | + | - | - | - | - | - | - | 3 | 7 |
| 35 | Qifengzhan | + | - | - | - | - | - | - | 3 | 7 |
| 36 | Lvhuangzhan | + | - | - | - | - | - | + | 3 | 3 |
| 37 | Zhen'gui'ai1 | + | - | - | - | - | - | - | 5 | 7 |
| 38 | Tesan'ai2 | + | - | - | - | - | - | - | 5 | 9 |
| 39 | Feng'aizhan1 | + | - | - | - | - | - | - | 3 | 5 |
| 40 | Qiangliuai | + | - | - | - | - | - | - | 9 | 9 |
| 41 | Changsizhan | + | - | - | - | - | - | - | 3 | 9 |
| 42 | Fengbazhan | - | - | - | - | - | - | - | 9 | 9 |
| 43 | Xianxiaozhan | + | - | - | - | - | - | - | 5 | 7 |
| 44 | Jingxian89 | + | - | - | - | - | - | - | 3 | 7 |
| 45 | Guichao2 | + | - | - | - | - | - | - | 9 | 9 |
| 46 | Texianzhan13 | + | - | - | - | - | - | - | 3 | 9 |
| 47 | Qiguizao25 | - | - | - | - | - | - | - | 5 | 7 |
| 48 | Xiaonongzhan | + | - | - | - | - | - | - | 9 | 9 |
| 49 | San’erai | + | - | - | - | - | - | - | 7 | 9 |
| 50 | Qidaizhan1 | + | - | - | - | - | - | - | 5 | 7 |
| 51 | Guangyinruanzhan | + | - | - | - | - | - | - | 5 | 9 |
| 52 | Huazhan | + | - | - | - | - | - | - | 7 | 7 |
| 53 | Guanghui998 | + | - | - | - | - | - | - | 7 | 7 |
| 54 | Yuehui613 | + | - | - | - | - | - | - | 5 | 7 |
| 55 | Guanghui128 | + | - | - | - | - | - | - | 7 | 9 |
| 56 | Guanghui308 | + | - | - | - | - | - | - | 5 | 7 |
| 57 | Guanghui615 | + | - | - | - | - | - | - | 5 | 7 |
| 58 | Guanghui3550 | + | - | - | - | - | - | - | 9 | 7 |
| 59 | Guanghui169 | + | - | - | - | - | - | - | 7 | 9 |
| 60 | Guanghui3618 | + | - | - | - | - | - | - | 7 | 7 |
| 61 | Guanghui122 | + | - | - | - | - | - | - | 3 | 9 |
| 62 | R7116 | - | - | - | - | - | - | - | 3 | 9 |
| 63 | Bing4114 | - | - | - | - | - | - | - | 5 | 5 |
| 64 | Guang 8B | + | - | - | - | - | - | - | 5 | 7 |
| 65 | Bo B | - | - | - | - | - | - | - | 9 | 9 |
| 66 | Qiu B | + | - | - | - | - | - | + | 9 | 9 |
| 67 | Wufeng B | + | - | - | - | - | - | - | 9 | 9 |
| 68 | Yuefeng B | - | - | - | - | - | - | + | 5 | 9 |
| 69 | Tianfeng B | + | - | - | - | - | - | - | 9 | 9 |
| 70 | Y58S | - | - | - | - | - | - | - | 5 | 7 |

Note: “+” and “-” represent positive and negative, respectively.

Table S2. Resistance level of the novel P/TGMS lines against rice blast in the natural blast nursery.

| Cropping-season | Tai S | Wo S | Yuejingsimiao2 (positive control) | Yueluzhan (negative control) |
| --- | --- | --- | --- | --- |
| 2019-L | 3 | 3 | 3 | 9 |
| 2019-E | 3 | 3 | 3 | 9 |
| 2018-L | 3 | 3 | 3 | 9 |
| 2018-E | 3 | 3 | 3 | 9 |
| 2017-L | 3 | 3 | 3 | 9 |
| 2017-E | 3 | 3 | 3 | 9 |

Table S3. Primers used in this study.

| Gene | Primer Name | Forward primer (5'→3') | Reverse primer (5'→3') |
| --- | --- | --- | --- |
|  |  |  |  |
| *Xa4* | RM224 | ATCGATCGATCTTCACGAGG | TGCTATAAAAGGCATTCGGG |
| *Xa7* | STSP3 | CAGCAATTCACTGGAGTAGTGGTT | CATCACGGTCACCGCCATCTCGGA |
| *Xa21* | pTA248 | AGACGCGGAAGGGTGGTTCCCGGA | AGACGCGGTAATCGAAAGATGAAA |
|  | U1/I2 | CGATCGGTATAACAGCAAAAC | TCTGATCATGCATGTTCTGTG |
| *Xa23* | RM206 | CCCATGCGTTTAACTATTCT | CTTTCCATCTATCCGTATGC |
|  | P23 | TGCCACAGCCAAAGTATGATGCT | TTGGCCCCTGCAGAGAAGGC |
| *Xa27* | Xa27-Co | TAGTGTCTAAATACAGGGACT | GAGTACTTTGCTCTGATGCTC |
| *xa34* | BGID36 | CGAGCACATCCTCCTCATTGCG | GTAGGTGGCTACGGCGAT |
| *Pi2* | S29742 | CAGTGAAACGAACGCTATG | AATAGGAAGGGTTGATGTTG |
|  | RM527 | GGCTCGATCTAGAAAATCCG | TTGCACAGGTTGCGATAGAG |
